# Mixed management in growing and finishing pigs: Differences between gender and their impacts on behavior, growth performance, and physiological parameters

**DOI:** 10.1371/journal.pone.0284481

**Published:** 2023-04-13

**Authors:** Angela Cristina da Fonseca de Oliveira, Leandro Batista Costa, Saulo Henrique Weber, Yuliaxis Ramayo-Caldas, Antoni Dalmau

**Affiliations:** 1 Graduate Program of Animal Science, Pontifícia Universidade Católica do Paraná –PUCPR, Curitiba, Paraná, Brazil; 2 Porcine Control and Evaluation, Institut de Recerca i Tecnologia Agroalimentàries–IRTA, Monells, Spain; University of Life Sciences in Lublin, POLAND

## Abstract

Mixing, a common management strategy used to regroup pigs, has been reported to impair individual performance and affect pig welfare because of the establishment of a new social hierarchy after regrouping. In this study we aimed to determine whether mixing management (non-mixed *vs*. mixed) and gender (gilts *vs*. barrows) affect the social and non-social behavior, performance, and physiological parameters of pigs. A total of 96 growing pigs (48 barrows and 48 females) were separated into two treatments: control (CT)—pigs that were mixed once during the growing-finishing period; and social stress (SS)—pigs that were mixed thrice during the growing-finishing period. We recorded social and non-social behaviors, injury score, performance, and physiological parameters during the experimental period. Data were grouped by the period, based on each mix performed, and overall values. The statistical analysis performed considered gender and treatment. For treatment, during period–II and III, the SS group presented the highest frequency of agonistic interactions (AI), stayed longer lying laterally (LL) and sternly (LS), and explored more enrichment material (ER) than the CT group. Furthermore, SS pigs presented the highest injury score in the ear, head, and middle and posterior regions. Compared to the females, the barrows spent more time at the electronic feed station and initiated most of the agonistic interactions during period–II, and they presented a higher injury score for the ear and head regions during period–III. In conclusion, repeated regrouping significantly affected social and feeding behavior without severely altering performance and physiological parameters. Furthermore, different patterns of social and feeding behavior, agonistic interactions, and injury scores between barrows and females were observed. This study provides an understanding of the impact of mixing management and gender differences on pigs, and this knowledge can be used to improve swine productivity and welfare.

## Introduction

Pig husbandry is recognized as a stressful production system [1]. The animals, from birth to slaughter, have to cope with multiple stressors such as early weaning that includes separation from the sow, handling and transportation, mixing with unfamiliar pigs, feed change, and exposure to different pathogens [2]. Among the farm practices, mixing is a common strategy used to regroup pigs according to weight to adjust group size, obtain uniform groups, and facilitate management [3]. Previous studies suggest that mixing impair individual performance and affect welfare due to the establishment of a new social hierarchy after regrouping [4, 5]. Management interventions available to producers do not successfully mitigate aggression but rather reduce severity and duration of aggression or delay its onset [6, 7].

In animal husbandry, gender is thought to be one of the most common factors that influence animal behavior [8, 9]; it affects the social structure in mice [9] and aggressive behavior in pigs [10]. Previous studies demonstrated that the time required to achieve social stability was lower for females than for males, suggesting behavioral differences between gilts and males near market weight [11, 12]. Furthermore, males do not only initiate more aggression than females but also win more fights [13]. However, the differences between pig behavior, performance, and physiological parameters, based on gender (barrows *vs*. females), after mixing remain unclear.

In view of the regrouping impacts [4, 5], the first hypothesis of the present study was that mixed pigs will display impaired social behavior (increase negative interactions, reduce positive interactions, and increased number of skin lesions); reduced non-social behaviors (time spent in lying, eating, drinking, exploring the pen, and enrichment material); and impaired performance and physiological parameters. Based on gender differences reported previously [8, 9], the second hypothesis of the present study was that females will display different patterns of social and non-social behavior after mixing (longer time spent lying, lower number of agonistic interactions, and lower number of skin lesions) compared to barrows. Considering that barrows are expected to be more involved in agonistic interactions, it is expected that they demonstrate poor performance and physiological parameters than females. Therefore, the objective of the present study was to evaluate whether mixing management (non-mixed *vs*. mixed) and gender (gilts *vs*. barrows) affect social and non-social behavior, growth performance, and physiological parameters in pigs.

## Materials and methods

### Ethics declarations

This research adhered to the legal requirements of the country, and the study was carried out according to all institutional guidelines. The procedures used were approved by the Comisión de Experimentación Animal de La Generalitat de Catalunya (protocol number—10329). The animals did not undergo any surgical procedure or management for which anesthesia and/or analgesia was required. At the end of the experiment, the animals were transported to a slaughterhouse and the slaughtering was performed following the European regulation 1099/2009. The housing conditions and management procedures followed those of the EU pig standards—COUNCIL DIRECTIVE 2008/120/EC of 18 December 2008.

### Animals and experimental design

A total of 96 growing pigs (48 barrows and 48 females; Duroc Commercial Line) were divided into eight pens and separated into two treatments, with four pens per treatment. Each experimental unit consisted of 12 animals (six barrows and six females per pen), with an initial average body weight (BW) of 18.63 ± 3.05 kg and a final average BW of 129.98 ± 10.04 kg. The pigs were grouped according to weight (pen from the lightest to the heaviest animals). The experiment lasted 157 days, divided into 30 days of adaptation period and 127 days of experimental protocol period.

### Housing conditions

The study was approved by the Institutional Animal Care and Use Committee (IACUC) of IRTA. Pigs were transported from a commercial farm to the experimental facilities of IRTA (Monells, Spain) and underwent a 30-day adaptation period prior to the start of the experiment. The housing conditions and management procedures followed those of the EU pig standards–COUNCIL DIRECTIVE 2008/120/EC of 18 December 2008. Enrichment material (chains and wood fixed to the wall) was made available in each pen during all the experimental phases (adaptation and growing-finishing period). The pens (5 m × 2.6 m) had a fully slatted floor, an electronic feeder system, and a nipple drinker. The unobstructed floor area available to each rearing pig was > 1 m^2^. The room was climate-controlled and the temperature was set to 19 ± 2°C with a light regime of 12 h light–dark cycle. The pigs received feed and water *ad-libitum*, throughout the experimental period. The feed was an isonutritive diet formulated according to the nutrient requirements of pigs for each respective period [14].

### Treatments

The experimental treatments were as follows: T1 –control group (CT), pigs that were mixed once during the growing-finishing period; and T2 –stress group (SS), pigs that were mixed three times during the growing-finishing period (social stress).

#### Social stress

**Fig 1:**
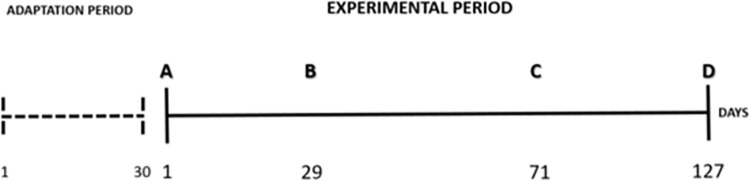
Experiment timeline. This study was conducted for 157 days, with 30 days for adaptation and 127 days for experimental protocol, following this timeline sequence: A: beginning of the experiment and first mix performed (distribution of control (CT) and stress (SS) groups); B: representation of the second mix performed (females’ mix); C: representation of the third mix performed (barrows’ mix); D: end of the experiment and pigs’ slaughter.

After the initial distribution according to treatments (day 0), considered the first mix performed, the SS group were further mixed twice at different times during the experimental period (Fig 1).

**Fig 2:**
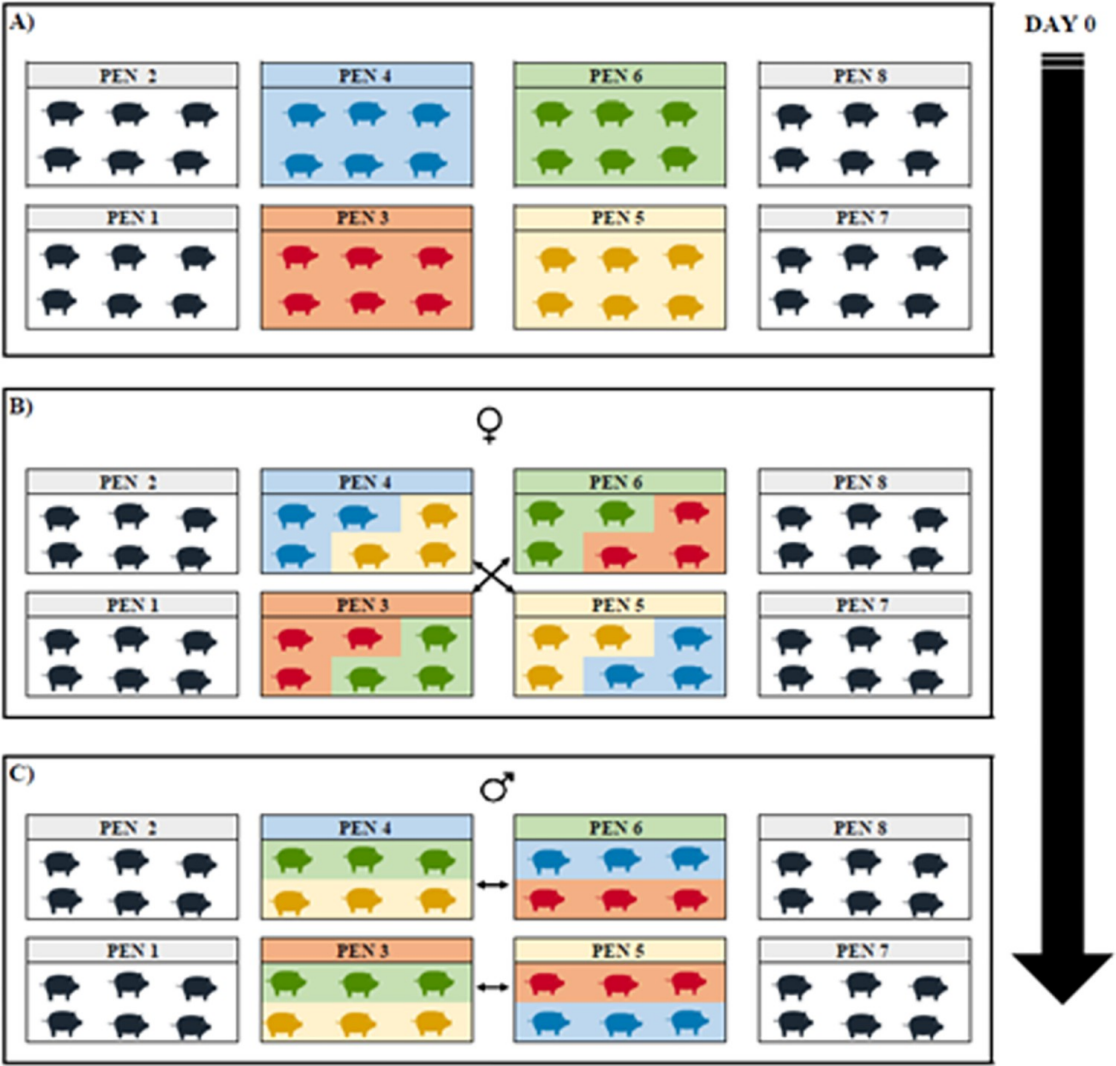
Stress factor related to the mixing scheme performed. A) Representation of the distribution performed at the beginning of the study (first mix). The gray pens represent the control group (pens 1, 2, 7 and 8). The colored pens represent the stress group (pens 3, 4, 5 and 6). B) Mix of females–representation of the second mix performed. The females in pen 3 switched places with the females in pen 6. The females in pen 4 switched places with the females in pen 5. The barrow group remained unchanged in all pens. C) Mix of barrows–representation of the third mix performed. The barrows in pen 3 switched places with the barrows in pen 5. The barrows in pen 4 switched places with the barrows in pen 6.

The second mix was in the fifth week of the experiment (day 29), and only the females switched places. Considering that all pens consisted of equal number of barrow and female animals, the resident barrows remained unchanged. The females in pen number 3 switched places with the females in pen number 6 and the females in pen number 4 switched places with the females in pen number 5 (Fig 2). The third mix was in the eleventh week of the study (day 71), and only barrows were switched; therefore, resident females remained unchanged. The barrows in the pen number 3 switched places with the barrows in the pen number 5 and the barrows in the pen number 4 switched places with the barrows in the pen number 6.

During the study, the pigs in the control pens remained unchanged. The study was divided into the following three periods for analysis of the results: period I–start of experimental period until the mixing of females (28 d); period II–mixing of females until the mixing of barrows (42 d); period III–mixing of barrows until the end of the experimental protocol (57 d).

### Performance and feeding behavior

The number of visits to the feeder, duration of the event–meal duration (s), and total feed consumption per event (kg) were recorded daily. Body weight (kg), average daily gain (kg), and average daily feed intake (kg) were recorded using weighing scale and electronic feeding stations. Each animal was individually weighed once a month, totaling four times during the experimental period. Based on these data, the average daily feed intake (ADFI), average daily gain (ADG), and feed conversion ratio (gain:feed; F:G) were determined.

### Sampling methods after mixing

The sampling methods applied in the present study were adapted from the report by [15] and the behavior aspects were according to Welfare Quality® Assessment Protocol for Pigs [16]. Based on the standard time of dominance establishment [17] the groups were directly observed during the first 72-hours after mixing. All observations during the entire experimental period were performed by two previously trained observers, to prevent the observers from having a bias effect on the results.

#### Agonistic interactions

Based on continuous event sampling, all occurrences of agonistic interaction were recorded within a sample period of 3 min [15] (Table 1). In the present study, an event of agonistic interaction was considered when an aggressive behavior that lasted longer than 1 s was initiated by one pig towards another. The end of an agonistic interaction was defined when one animal, that is, the loser showed a submissive behavior and the two opponents were separated for at least 5 s [18]. An agonistic interaction was defined as indecisive if no clear submissive behavior was detected even though the animals were separated from each other for at least 5 s. For each agonistic interaction, the author, receiver, winner, loser, and area of the pen in which the interaction occurred were recorded. Only those agonistic interactions between two animals, with a clear author and receiver as well as a clear winner and loser were used for further analyses.

**Table 1:** Definition of the ethological patterns used.

Term	Definition
**Agonistic interaction (AI)**	Physical contact between individuals, with or without reaction on the part of the receiver, leading to an attack (unilateral action; the receiver does not bite back) or a fight (bilateral action; the receiver counter-attack).
**Submissive behavior**	Body-turning (receiver pig turns whole body 180 degrees to protect head and ear), usually accompanied by an ear bite and flight response by the attacked pig.
**Author**	Attacking pig.
**Receiver**	Pig being attacked.
**Winner**	A pig is considered a winner when the other individual involved in the agonistic interaction shows a flight and/or submissive behavior. The winner may be the individual who started the AI or not.
**Loser**	The loser of an agonistic interaction is the one who showed an escape movement during the AI. The loser may be the individual who initiated the AI or not.

McGlone JJ. A Quantitative Ethogram of Aggressive and Submissive Behaviors in Recently Regrouped Pigs. J Anim Sci. 1985; 61(3): 556–566. https://doi.org/10.2527/jas1985.613556x

### Scan sampling

After the agonistic interactions were recorded (3 min), an instantaneous sampling was carried out to record the primary activities of the animals (1 min). Instantaneous sampling or scan sampling is a technique in which the observer records the current activity of an individual at preselected moments in time; it is a sample of states and not events (e.g., in a minute throughout the day) [15]. The behaviors recorded were the following: eating, exploring the ambient of the pen, exploring the environmental enrichment material (chains and wood fixed to the wall), lying (sternly or laterally), drinking, positive and negative interactions, and others (any behavior that does not fit into the activities described above). Positive interactions were defined as sniffing, nosing, licking, and moving gently away from the animal without aggressive or flight reaction from the individual.

At the end of the evaluations, the observer moved to the next pen. The same was done until all the pens were evaluated. These evaluations were conducted for a total of two consecutive hours in the morning (0900–1100 h) and two consecutive hours in the afternoon (1400–1600 h). A total of 12 continuous event sampling and 12 scan sampling per pen/day were completed. At the end of three days, each pen was evaluated 36 times for continuous event sampling and 36 times for scan sampling, adding up to a total of 288 observations per treatment per period.

#### Injury score

At the end of each day (1600 h), during the 72-hours after mixing, the number of injuries were counted. Injuries on the body were visually and individually assessed via inspection of two sides of the body (right and left), and the number of injuries per area (body region of the pig) was recorded. The four areas considered were as follows: (1) front portion (head and ears); (2) medial portion; (3) posterior portion (rear and tail); and (4) legs. The injuries considered were scratches (surface penetration on the epidermis) or wounds (penetration of the muscle tissue) [16]. Each day, only fresh lesions were computed to avoid overestimation of results. A lesion was considered only if it had a minimum length of minimum 2 cm.

### Physiological measures

To evaluate the physiological parameters of the pigs, blood and hair samples were collected. Blood samples were collected twice, one at the end of the adaptation period (basal values–day 27) and another a day before slaughtering (final values–day 127). Once the hair cortisol analysis captures long-term cortisol secretion, the hair samples were collected just once at the end of the experimental period (day 127). The authors opted for the analysis of cortisol in hair and not blood or feces samples, because hair cortisol is a unique and non-invasive means for capturing of long-term cortisol secretion [19]. All animals were sampled.

#### Blood samples

Blood samples were obtained from the jugular vein after restraining the pig by the snout with a loop. The animal was maintained at standing position and head at a 30-degree angle. Subsequently, 8 mL of blood was collected and aseptically stored in tubes containing the anticoagulant ethylenediaminetetraacetic acid (EDTA; Becton Dickinson, USA) for analysis of blood count (hematocrit, hemoglobin, red blood cell, mean corpuscular volume, platelets, leukocytes, eosinophils, basophils, lymphocytes, monocytes, neutrophils, and neutrophil/lymphocyte ratio).

#### Hair cortisol samples

For cortisol analysis, hair samples (3.0 cm—hair length) were obtained from the loin part of the dorso-lumbar (L) region, taking advantage of the restrains provided by the weighing scale during regular weighing. Pigs were gently accompanied to the scales, which had a two-door system for access and exit. Hair was collected by shaving close to the skin with clippers without removing the root of the hair, and caution was taken not to include hair follicle in the sample. After sampling, hair was stored at ambient temperature inside hermetically sealed bags until analysis. Cortisol extraction was performed following the method described by [20], with a few modifications. First, approximately 150 mg of hair was washed twice in 3 mL of 99.5% isopropanol for 30 s to eliminate contaminants that could interfere with the determination. Hair was then allowed to dry overnight in an airflow hood. The following day, samples were finely minced using surgical scissors until hair segments were a maximum of 0.3 cm in length.

For cortisol extraction, 1 mL of 99.5% methanol was added to approximately 50 mg of powdered hair and then incubated at 37°C for 17 hours with slow rotation. Subsequently, the sample was spun in a microcentrifuge for 30 s at 5000 rpm. At the end of the extraction, the sample was centrifuged and 0.6 mL of the supernatants were finally dried using a vacuum centrifuge and stored at– 20°C. The dry extract was reconstituted in phosphate buffer solution from the assay kit. Hair cortisol concentration was assessed using a High Sensitivity Salivary Cortisol enzyme immunoassay (EIA) kit (Salimetrics, State College, PA, USA) following the manufacturer’s instructions. All cortisol analyses were performed in the IRTA–Institute of Agrifood Research and Technology (Barcelona, Spain).

### Statistical analysis

Statistical analyses were performed using STATGRAPHICS Centurion XVI statistical Software^Ⓡ^, Version 16.11 and all data were compared according to gender (barrow *vs*. female) and treatment (CT *vs*. SS). Data from parametric variables were compared using analysis of variance (ANOVA-Type III) followed by Tukey’s test when homogeneity of variance was observed (Levene’s test). Data that did not show normality (Shapiro–Wilk) or homogeneity of variance (Levene) were subjected to a Wilcoxon test. The results are presented as mean ± standard error. Three statistical analyzes were used for non-parametric data, depending on the type and variation of the data obtained. Frequency data such as number of visits to feeder, quadrant occupation, scan sampling (frequency of observed behaviors), and injury score, were compared using Mann–Whitney (Wilcoxon) W-test. Nonparametric data from binary analyzes (yes or no) were compared using Kendall’s tau-b (τb) correlation coefficient. In addition, the frequencies of agonistic interaction according to treatment and gender were compared two by two using chi-square test. All the non-parametric data are presented as the medians and minimum-maximum values and the description of each test used is presented as footnote for each Table. P-values ≤ 0.05 were considered significant.

## Results

### Performance and feeding behavior

The overall results of the performance and feeding behavior (127 d of experimentation) of pigs according to treatment (CT *vs*. SS) and gender (female *vs*. barrow) are shown in Table 2. No influence of treatment or gender was observed for the variables analyzed (*p* > 0.05).

**Table 2:** Overall data of the performance (mean ± SE) and feeding behavior (median, min—max) of growing and finishing pigs, according to treatment and gender.

	Treatment			Gender		
Item	CT	SS	*p*-value	Female	Barrow	*p*-value
**^1^One-way ANOVA**						
**IBW (Kg)**	26.33 ± 0.62	25.93 ± 0.59	0.642	26.68 ± 0.60	25.58 ± 0.62	0.203
**FBW (Kg)**	130.22 ± 1.55	129.66 ± 1.48	0.796	131.11 ± 1.49	128.77 ± 1.53	0.278
**ADG (Kg)**	0.88 ± 0.01	0.88 ± 0.01	0.911	0.88 ± 0.01	0.87 ± 0.01	0.540
**ADFI (Kg)**	2.55 ± 0.05	2.48 ± 0.04	0.283	2.55 ± 0.04	2.48 ± 0.05	0.297
**F:G**	2.90 ± 0.04	2.83 ± 0.04	0.284	2.89 ± 0.04	2.84 ± 0.04	0.443
**ATES (h)**	1.00 ± 0.03	1.04 ± 0.03	0.282	0.99 ± 0.03	1.05 ± 0.03	0.098
**^2^Mann–Whitney: W-Test**						
**NVF (n)**	10.99 (6.4–20.4)	10.64 (4.7–18.2)	0.750	10.82 (4.7–18.2)	10.88 (6.4–20.4)	0.869

CT: pigs that were mixed once during the growing-finishing period; SS: pigs that were mixed three times during the growing-finishing period.

Period of 127 d under control.

IBW: initial body weight; FBW: final body weight; ADG: average daily gain; ADFI: average daily feed intake; F:G: feed to gain ratio; ATES: average daily time at the electronic station; NVF: number of visits to the feeder.

^1^Parametric data are presented as mean ± standard error and compared using ANOVA-Type III, followed by Tukey’s test when homogeneity of variance (Levene’s test) was observed.

^2^Nonparametric data resulting from scores are presented as median (minimum–maximum), and Mann–Whitney (Wilcoxon) W-test was used to compare the medians of the two samples.

Table 3 shows the results of performance and feeding behavior according to the period of mixing (stress factor applied). There was no statistical difference between treatments for IBW, FBW, ADG, ADFI, F:G, and NVF. However, for ATES (period I), the SS group spent more time at the electronic feed station than the CT group (1.27 h *vs*. 1.10 h) (*p* ≤ 0.05). For ATES (periods II and III), no statistical difference was found. For genders (Female *vs*. Barrow), there was no statistical difference for the variables IBW, FBW, ADG, F:G, and NVF. For the variables ADFI and ATES, females had a higher ADFI during period III (3.27 kg *vs*. 3.07 kg) and the barrows spent more time at the electronic feed station than the females during period II (1.30 h *vs*. 1.12 h) (*p* ≤ 0.05).

**Table 3:** Data of performance (mean ± SE) and feeding behavior (median, min—max) of growing and finishing pigs according to treatment, gender, and period of mixing.

		Treatment			Gender		
Item	Period	CT	SS	*p*-value	Female	Barrow	*p*-value
**^1^One-way ANOVA**							
**IBW (Kg)**	**I**	26.33 ± 0.62	25.93 ± 0.59	0.642	26.68 ± 0.60	25.58 ± 0.62	0.203
	**II**	41.72 ± 0.64	41.35 ± 0.61	0.681	42.13 ± 0.61	40.94 ± 0.63	0.183
	**III**	75.56 ± 1.03	75.08 ± 0.98	0.735	76.01 ± 0.99	74.63 ± 1.02	0.336
**FBW (Kg)**	**I**	41.72 ± 0.64	41.35 ± 0.61	0.681	42.13 ± 0.61	40.94 ± 0.63	0.183
	**II**	75.56 ± 1.03	75.08 ± 0.98	0.735	76.01 ± 0.99	74.63 ± 1.02	0.336
	**III**	130.22 ± 1.55	129.66 ± 1.48	0.796	131.11 ± 1.49	128.77 ± 1.53	0.278
**ADG (Kg)**	**I**	0.81 ± 0.01	0.81 ± 0.01	0.910	0.81 ± 0.01	0.81 ± 0.01	0.801
	**II**	0.83 ± 0.01	0.82 ± 0.01	0.855	0.83 ± 0.01	0.82 ± 0.01	0.769
	**III**	1.03 ± 0.02	1.03 ± 0.02	0.921	1.04 ± 0.02	1.02 ± 0.02	0.431
**ADFI (Kg)**	**I**	1.51 ± 0.06	1.54 ± 0.06	0.734	1.58 ± 0.06	1.46 ± 0.06	0.169
	**II**	2.49 ± 0.07	2.40 ± 0.07	0.368	2.39 ± 0.07	2.50 ± 0.07	0.259
	**III**	3.22 ± 0.07	3.12 ± 0.07	0.307	3.27 ± 0.07 A	3.07 ± 0.07 B	0.042
**F:G**	**I**	1.87 ± 0.09	1.92 ± 0.08	0.663	1.98 ± 0.08	1.82 ± 0.09	0.184
	**II**	3.00 ± 0.07	2.93 ± 0.07	0.478	2.89 ± 0.07	3.04 ± 0.07	0.140
	**III**	3.12 ± 0.05	3.04 ± 0.05	0.315	3.15 ± 0.05	3.01 ± 0.05	0.073
**ATES (h)**	**I**	1.10 ± 0.04 b	1.27 ± 0.04 a	0.005	1.20 ± 0.04	1.17 ± 0.04	0.588
	**II**	1.21 ± 0.04	1.21 ± 0.04	0.963	1.12 ± 0.04 B	1.30 ± 0.04 A	0.003
	**III**	0.85 ± 0.03	0.88 ± 0.03	0.489	0.86 ± 0.03	0.87 ± 0.03	0.695
**^2^Mann–Whitney: W-Test**							
**NVF (n)**	**I**	332 (24–768)	356 (160–718)	0.475	352 (160–768)	342 (24–718)	0.634
	**II**	519 (299–1179)	518 (152–916)	0.175	501.5 (189–916)	522.5 (152–1179)	0.881
	**III**	421 (238–706)	399 (201–871)	0.657	386.5 (205–871)	408.5 (201–676)	0.672

CT: pigs that were mixed once during the growing-finishing period; SS: pigs that were mixed three times during the growing-finishing period.

Period: I–start of experimental control until the mixing of females (28 d); II–mixing of females until the mixing of barrows (42 d); III–mixing of barrows until the end of the experimental protocol (57 d).

IBW: initial body weight; FBW: final body weight; ADG: average daily gain; ADFI: average daily feed intake; F:G: feed to gain ratio; ATES: average daily time at the electronic station; NVF: number of visits to the feeder.

^1^Parametric data are presented as mean ± standard error and compared using ANOVA-Type III, followed by Tukey’s test when homogeneity of variance (Levene’s test) was observed.

^2^Nonparametric data resulting from scores are presented as median (minimum–maximum), and Mann–Whitney (Wilcoxon) W-test was used to compare the medians of the two samples.

^A,b^ Different letters on the same row for treatment represent differences between means using ANOVA followed by Tukey-test (*p* ≤ 0.05).

^A,B^ Different letters on the same row for gender represent differences between means using ANOVA followed by Tukey-test (*p* ≤ 0.05).

### Sampling methods after mixing

Data obtained from continuous observation are presented in Table 4. No statistical differences were found between gender and treatment for the number for agonistic interactions (NAI) during period–I. During period–II, and the SS group presented a higher frequency of NAI than the control group (*p ≤* 0.05). The barrows initiated most of the interactions and the females were more of receptors (*p* ≤ 0.05). For period–III, the highest number of agonistic interactions was observed in the SS group (*p* ≤ 0.05), but no differences were observed between genders. The barrows and females had the same number of author and receiver position.

**Table 4:** Number of agonistic interactions of growing and finishing pigs under control (CT) and stress (SS) conditions according to treatment and gender.

Item	*Treatment*		*Gender*	
	CT	SS	Female	Barrow
**Period–I**				
**Author**	98	79	94	83
**Receiver**	98	79	100	77
**Period–II**				
**Author**	32 b	85 a	44 B	73 a
**Receiver**	32 b	85 a	77 A	40 B
**Period–III**				
**Author**	6 b	45 a	28	23
**Receiver**	6 b	45 a	21	30

CT: pigs that were mixed once during the growing-finishing period; SS: pigs that were mixed three times during the growing-finishing period.

Period: I–start of experimental control until the mixing of females (28 d); II–mixing of females until the mixing of barrows (42 d); III–mixing of barrows until the end of the experimental protocol (57 d).

^a,b^ Different letters on the same row for treatment represent differences between observed frequencies according to chi-squared test *(p ≤* 0.05).

^A,B^ Different letters on the same row for gender represent differences between observed frequencies according to chi-squared test (*p* ≤ 0.05).

### Scan sampling

Data obtained from scan sampling (quadrant occupation and observed behaviors) are presented in Tables 5 and 6, respectively. During period—I, the animals in the CT group occupied the Q-3 (quadrant located laterally, located at the background of the pen) more, whereas the animals in the SS group occupied the Q-1 (quadrant at the entrance to the pen) more (*p* ≤ 0.05). No differences were observed between groups for the occupation of Q-2. During period—II, the pattern observed was similar to that observed during period—I for quadrants Q-1 and Q-3. However, statistical differences were observed for Q-2. The SS group occupied the Q-2 more often than the CT group *(p ≤* 0.05). During period—III, the SS group occupied Q-1 more than the CT group (*p* ≤ 0.05). No statistical differences were observed for the occupation of the Q-2 and Q-3 quadrants.

**Table 5:** Data from scan sampling method on quadrant occupation for growing and finishing pigs (presented in median / min—max).

	Treatment		
Item	CT	SS	*p*-value
**Period—I (n)**			
**QO– 1**	3 (0–10) b	6 (0–11) a	0.000
**QO– 2**	4 (0–10)	4 (1–8)	0.646
**QO– 3**	4 (0–12) a	2 (0–10) b	0.000
**Period—II (n)**			
**QO– 1**	5 (0–9) b	5 (0–10) a	0.004
**QO– 2**	4 (1–7) b	4 (1–8) a	0.010
**QO– 3**	3 (0–7) a	2 (0–8) b	0.022
**Period—III (n)**			
**QO– 1**	4 (2–8) b	5 (1–9) a	0.005
**QO– 2**	3 (0–8)	3 (1–8)	0.534
**QO– 3**	3 (0–7)	3 (0–7)	0.227

CT: pigs that were mixed once during the growing-finishing period; SS: pigs that were mixed three times during the growing-finishing period.

Period: I—start of experimental control until the mixing of females (28 d); II—mixing of females until the mixing of barrows (42 d); III—mixing of barrows until the end of the experimental protocol (57 d).

QO—1: quadrant at the entrance to the pen; QO—2: central quadrant, where the feeders were positioned; and QO—3: quadrant located laterally and at the background of the pen.

^a,b^ Different letters on the same row for treatment represent differences between medians according to Mann–Whitney (Wilcoxon) W-test (*p* ≤ 0.05).

**Table 6:** Frequency of observed behaviors of pigs at growing and finishing phases obtained through the scan sampling method and presented as MD (median / min—max) and absolute value.

		Treatment		
Item	Period	CT	SS	*p*-value
**LL—Lying laterally (n)**				
** **	**I**	1 (0–11)	0 (0–9)	0.795
** **	**II**	1 (0–5) b	1 (0–8) a	0.022
** **	**III**	2 (0–8) b	2 (0–10) a	0.036
**LS—Lying sternly (n)**				
** **	**I**	5 (0–11)	5 (0–10)	0.738
** **	**II**	3 (0–8) b	4 (0–8) a	0.039
** **	**III**	4 (0–9) b	5 (0–11) a	0.000
**EX—Exploring (n)**	** **			
** **	**I**	1 (0–7)	1 (0–8)	0.239
** **	**II**	2 (0–9)	2 (0–9)	0.067
** **	**III**	1 (0–6) a	0 (0–5) b	0.000
**ER—Exploring enrichment material (n)**				
** **	**I**	0 (0–2)	0 (0–1)	0.531
** **	**II**	0 (0–2) b	0 (0–3) a	<0.001
** **	**III**	0 (0–1)	0 (0–4)	0.263
**E—Eating (n)**	** **			
** **	**I**	1 (0–1)	1 (0–2)	0.901
** **	**II**	1 (0–1)	1 (0–1)	0.467
** **	**III**	1 (0–1) a	1 (0–1) b	0.045
**DR—Drinking (n)**	** **			
** **	**I**	0 (0–1) a	0 (0–2) b	0.020
** **	**II**	0 (0–1)	0 (0–1)	0.876
** **	**III**	0 (0–2)	0 (0–1)	0.369
**PI—Positive interaction (n)**				
** **	**I**	0 (0–4)	0 (0–4)	0.161
** **	**II**	0 (0–5)	0 (0–4)	0.198
** **	**III**	0 (0–4)	0 (0–4)	0.310
**NI—Negative interaction (n)**				
** **	**I**	0 (0–2)	0 (0–4)	0.714
** **	**II**	0 (0–2) b	0 (0–6) a	0.049
** **	**III**	0 (0–2) b	0 (0–4) a	0.005
**O—Others (n)**	** **			
** **	**I**	1 (0–11)	1 (0–10)	0.450
** **	**II**	2 (0–8) a	2 (0–8) b	0.001
** **	**III**	2 (0–5) a	1 (0–6) b	0.005

CT: pigs that were mixed once during the growing-finishing period; SS: pigs that were mixed three times during the growing-finishing period.

Period: I—start of experimental control until the mixing of females (28 d); II—mixing of females until the mixing of barrows (42 d); III—mixing of barrows until the end of the experimental protocol (57 d).

^a,b^ Different letters on the same row for treatment represent differences between medians according to Mann–Whitney (Wilcoxon) W-test (*p* ≤ 0.05).

Regarding the frequency of behaviors (Table 6) during period—I, the CT group spent more time drinking compared to the SS group (*p* ≤ 0.05). For period—II, compared to the CT group (*p* ≤ 0.05), the SS group presented the highest frequency for lying (laterally—LL and sternly—LS), exploring of enrichment material (ER), and agonistic interactions (AI), and the lowest frequency for other (O) behaviors. For period–III, similar results were observed for LL, LS, and NI, that is, they were higher in SS group than in CT group. In contrast, during the same period, the opposite was observed for exploring (EX), eating (E), and O behaviors (*p* ≤ 0.05). No differences were observed for positive interactions (PI) during the three periods.

### Injury score

Table 7 shows the observed injury score data. Based on treatment, CT group presented the highest score for RH (right head) lesions compared to SS group during period–I (*p* ≤ 0.05). No differences were found for the other regions. For period–II, SS group presented the highest score for LE, LH, LM, RE, RH, RM, and RPo regions compared to the CT group. (*p* ≤ 0.05). Similar result was observed during period–III for LE, LH, RE, and RH regions (*p* ≤ 0.05). No differences were observed for the other variables. Based on gender, the only differences observed were during period—III, in which the barrows had a higher lesion score for regions LE and LH than the females (*p* ≤ 0.05).

**Table 7:** Analysis of fresh lesion counts at different regions of growing and finishing pigs during 72 hours after mixing. Values presented as MD (median / min—max) according to treatment and gender.

	Treatment			Gender		
Item	CT	SS	*p*-value	Female	Barrow	*p*-value
**LE—Left ear (n)**						
**I**	0 (0–6)	0 (0–5)	0.307	0 (0–6)	0 (0–5)	0.778
**II**	0 (0–3) b	0 (0–6) a	0.000	0 (0–6)	0 (0–5)	0.855
**III**	0 (0–1) b	0 (0–5) a	0.000	0 (0–4) b	0 (0–5) a	0.003
**LH—Left head (n)**						
**I**	0 (0–8)	0 (0–3)	0.255	0 (0–5)	0 (0–8)	0.775
**II**	0 (0–6) b	0 (0–10) a	0.000	0 (0–10)	0 (0–10)	0.378
**III**	0 (0–0) b	0 (0–3) a	0.000	0 (0–3) B	0 (0–3) A	0.006
**LM—Left middle (n)**						
**I**	0 (0–7)	0 (0–2)	0.293	0 (0–7)	0 (0–4)	0.818
**II**	0 (0–4) b	0 (0–6) a	0.000	0 (0–5)	0 (0–6)	0.243
**III**	0 (0–0)	0 (0–1)	0.093	0 (0–1)	0 (0–1)	0.552
**LPo—Left posterior (n)**						
**I**	0 (0–2)	0 (0–4)	0.635	0 (0–4)	0 (0–2)	0.594
**II**	0 (0–2)	0 (0–5)	0.621	0 (0–3)	0 (0–5)	0.963
**III**	0 (0–0)	0 (0–2)	0.172	0 (0–2)	0–0–1)	0.996
**LPa—Left paws (n)**						
**I**	-	-	-	-	-	-
**II**	0 (0–1)	0 (0–1)	0.976	0 (0–1)	0 (0–1)	0.984
**III**	0 (0–0)	0 (0–1)	0.337	0 (0–0)	0 (0–1)	0.316
**RE—Right ear (n)**						
**I**	0 (0–5)	0 (0–5)	0.994	0 (0–5)	0 (0–5)	0.882
**II**	0 (0–3) b	0 (0–10) a	0.000	0 (0–4)	0 (0–10)	0.120
**III**	0 (0–1) b	0 (0–6) a	0.000	0 (0–3)	0 (0–6)	0.174
**RH—Right head (n)**						
**I**	0 (0–4) a	0 (0–3) b	0.024	0 (0–4)	0 (0–3)	0.544
**II**	0 (0–4) b	0 (0–8) a	0.000	0 (0–8)	0 (0–7)	0.489
**III**	0 (0–1) b	0 (0–5) a	0.000	0 (0–5)	0 (0–3)	0.304
**RM—Right middle (n)**						
**I**	0 (0–3)	0 (0–3)	0.590	0 (0–3)	0 (0–3)	0.325
**II**	0 (0–8) b	0 (0–13) a	0.001	0 (0–7)	0 (0–13)	0.141
**III**	-	-	-	-	-	-
**RPo—Right posterior (n)**						
**I**	0 (0–3)	0 (0–4)	0.847	0 (0–4)	0 (0–4)	0.218
**II**	0 (0–2) b	0 (0–3) a	0.002	0 (0–3)	0 (0–3)	0.981
**III**	0 (0–0)	0 (0–3)	0.337	0 (0–0)	0 (0–3)	0.316
**RPa—Right paws (n)**						
**I**	-	-	-	-	-	-
**II**	0 (0–1)	0 (0–1)	0.972	0 (0–1)	0 (0–1)	0.980
**III**	-	-	-	-	-	-

CT: pigs that were mixed once during the growing-finishing period; SS: pigs that were mixed three times during the growing-finishing period.

Period: I—start of experimental control until the mixing of females (28 d); II—mixing of females until the mixing of barrows (42 d); III—mixing of barrows until the end of the experimental protocol (57 d).

^a,b^ Different letters on the same row for treatment represent differences between medians according to Mann–Whitney (Wilcoxon) W-test (*p* ≤ 0.05).

^A,B^ Different letters on the same row for gender represent differences between medians according to Mann–Whitney (Wilcoxon) W-test (*p* ≤ 0.05).

### Physiological measures

Baseline and final average blood count and cortisol (only final) data are present in Tables 8 and 9, respectively according to treatment and gender. With regard to treatments, there was no statistical difference between treatments (CT and SS) for the initial blood samples. However, for the final samples, the CT group presented higher values for Seg:Limf (1.24 *vs*. 1.04) compared to the SS group (*p* ≤ 0.05). The other variables of blood count and cortisol in the hair showed no statistical difference for the final samples.

**Table 8:** Initial and final average blood count (mean ± SE) and cortisol samples (mean ± SE) of growing and finishing pigs according to treatment.

		Basal Values			Final Values		
^1^Item	-	CT	SS	*p*-value	CT	SS	*p*-value
** CBC–Complete Blood Count**	** **						
**HTC**	**%**	37.26 ± 0.49	36.82 ± 0.49	0.52	41.66 ± 0.67	42.13 ± 0.64	0.61
**HMB**	**g/dL**	10.46 ± 0.11	10.39 ± 0.11	0.67	13.01 ± 0.21	12.97 ± 0.20	0.87
**HMT (10** ^**6**^ **)**	**/μL**	6.21 ± 0.06	6.22 ± 0.06	0.94	7.12 ± 0.12	7.16 ± 0.12	0.80
**MCV**	**fL**	60.04 ± 0.68	59.28 ± 0.67	0.43	58.69 ± 0.66	59.03 ± 0.63	0.70
**MCH**	**pg**	16.88 ± 0.19	16.74 ± 0.19	0.60	18.31 ± 0.18	18.18 ± 0.18	0.61
**MCHC**	**g/dL**	28.16 ± 0.28	28.31 ± 0.28	0.70	31.22 ± 0.14	30.84 ± 0.14	0.07
**PLT (10** ^**5**^ **)**	**/μL**	3.78 ± 0.25	3.86970 ± 0.25	0.80	2.31 ± 0.14	2.64 ± 0.14	0.10
**WBC—White Blood Cells**	** **						
**LKC (10** ^**3**^ **)**	**%**	14.95 ± 0.40	15.53 ± 0.40	0.31	16.09 ± 0.61	16.82 ± 0.59	0.39
**EOS (10** ^**2**^ **)**	**/μL**	3.39 ± 0.54	4.34 ± 0.51	0.21	5.28 ± 0.50	6.07 ± 0.49	0.26
**BAS (10** ^**2**^ **)**	**/μL**	2.33 ± 0.65	2.01 ± 0.63	0.73	1.66 ± 0.30	2.01 ± 0.42	0.65
**LYM (10** ^**2**^ **)**	**/μL**	58.37 ± 2.76	58.38 ± 2.73	1.00	72.33 ± 5.18	84.11 ± 4.96	0.10
**MON (10** ^**2**^ **)**	**/μL**	3.77 ± 0.75	4.46 ± 0.70	0.51	3.48 ± 0.74	4.05 ± 0.66	0.56
**SEG (10** ^**2**^ **)**	**/μL**	85.54 ± 3.30	89.62 ± 3.26	0.38	81.37 ± 2.80	75.64 ± 2.68	0.14
**SEG:LIMF**	**- **	1.62 ± 0.11	1.70 ± 0.11	0.61	1.24 ± 0.07 a	1.04 ± 0.07 b	0.04
**CORTISOL**	** μg**	-	-	-	0.05 ± 0.001	0.05 ± 0.001	0.32

CT: pigs that were mixed once during the growing-finishing period; SS: pigs that were mixed three times during the growing-finishing period.

Period of 127 d under control.

^1^HTC: Hematocrit; HMB: Hemoglobin; HMT: Hematies; MCV: Mean corpuscular volume; MCH: Mean corpuscular hemoglobin; MCHC: Mean corpuscular hemoglobin concentration; PLT: Platelets LKC: leucocytes; EOS: Eosinophils; BAS: Basophils; LYM: Lymphocytes; MON: Monocytes; SEG: neutrophils; SEG:LYMF: neutrophil/lymphocyte ratio.

^a,b^ Different letters on the same row for treatment; final values represent differences between means according to ANOVA followed by Tukey-test (*p* ≤ 0.05).

**Table 9:** Initial and final average blood count (mean ± SE) and cortisol samples (mean ± SE) of growing and finishing pigs according to gender.

		Basal Values			Final Values		
^1^Item	-	Female	Barrow	*p*-value	Female	Barrow	*p*-value
**CBC–Complete Blood Count**	** **						
**HTC**	**%**	37.19 ± 0.49	36.90 ± 0.49	0.67	41.03 ± 0.65	42.76 ± 0.66	0.07
**HMB**	**g/dL**	10.42 ± 0.11	10.43 ± 0.11	0.98	12.70 ± 0.20 B	13.28 ± 0.20 A	0.04
**HMT (10** ^**6**^ **)**	**/μL**	6.15 ± 0.06	6.29 ± 0.06	0.11	7.00 ± 0.12	7.28 ± 0.12	0.12
**MCV**	**fL**	60.62 ± 0.67 a	58.71 ± 0.68 b	0.05	58.73 ± 0.64	58.99 ± 0.65	0.78
**MCH**	**pg**	17.01 ± 0.19	16.60 ± 0.19	0.13	18.20 ± 0.18	18.30 ± 0.18	0.68
**MCHC**	**g/dL**	28.13 ± 0.28	28.34 ± 0.28	0.60	30.99 ± 0.14	31.06 ± 0.14	0.74
**PLT (10** ^**5**^ **)**	**/μL**	3.90 ± 0.25	3.74 ± 0.26	0.65	2.64 ± 0.14	2.32 ± 0.14	0.10
**WBC—White Blood Cells**							
**LKC (10** ^**3**^ **)**	**%**	15.67 ± 0.40	14.81 ± 0.40	0.13	16.67 ± 0.60	16.24 ± 0.60	0.61
**EOS (10** ^**2**^ **)**	**/μL**	4.36 ± 0.50	3.37 ± 0.54	0.19	5.12 ± 0.50	6.23 ± 0.49	0.12
**BAS (10** ^**2**^ **)**	**/μL**	2.43 ± 0.70	1.90 ± 0.58	0.57	1.68 ± 0.32	1.96 ± 0.46	0.71
**LYM (10** ^**2**^ **)**	**/μL**	59.68 ± 2.73	57.07 ± 2.76	0.50	81.89 ± 5.07	74.56 ± 5.08	0.31
**MON (10** ^**2**^ **)**	**/μL**	4.19 ± 0.68	4.04 ± 0.77	0.88	4.06 ± 0.69	3.47 ± 0.71	0.56
**SEG (10** ^**2**^ **)**	**/μL**	89.71 ± 3.26	85.45 ± 3.30	0.36	77.37 ± 2.74	79.64 ± 2.74	0.56
**SEG:LYMF**	** -**	1.61 ± 0.11	1.70 ± 0.11	0.57	1.09 ± 0.07	1.19 ± 0.07	0.31
**CORTISOL**	**μg **	-	-	-	0.05 ± 0.0009	0.05 ± 0.0010	0.43

Period of 127 d under control.

^1^HTC: Hematocrit; HMB: Hemoglobin; HMT: Hematies; MCV: Mean corpuscular volume; MCH: Mean corpuscular hemoglobin; MCHC: Mean corpuscular hemoglobin concentration; PLT: Platelets LKC: leucocytes; EOS: Eosinophils; BAS: Basophils; LYM: Lymphocytes; MON: Monocytes; SEG: neutrophils; SEG:LYMF: neutrophil/lymphocyte ratio.

^a,b^ Different letters on the same row for gender; basal values represent differences between means according to ANOVA followed by Tukey-test (*p* ≤ 0.05).

^A,B^ Different letters on the same row for gender; final values represent differences between means according to ANOVA followed by Tukey-test (*p* ≤ 0.05).

With respect to gender (Table 9), the females presented higher values of MCV for basal samples compared to the barrows (*p* ≤ 0.05). For final samples, the barrows presented the highest hemoglobin count (*p* ≤ 0.05). There was no difference for the other variables (*p* ≤ 0.05).

## Discussion

### Performance and feeding behavior

Mixing is a common management strategy used to regroup pigs according to BW or size to decrease BW variability and facilitate farm management practices [3]. Previous studies suggest that mixing impair individual performance within a pen and affect pig welfare because of the establishment of a new social hierarchy after regrouping [4, 5]. The social stressors involved in regrouping could affect growth in a very short term or over an extended period. A reduction in growth during the first few days suggest increased metabolic response that interferes with weight gain or a behavioral change such as failure to eat or drink. Reductions in weight gain that occur several days after regrouping may be due to physiological responses to stress such as elevated corticosteroids, suppressed immune response, or continuing behavior changes such as reduced feed intake or excessive activity [21].

According to outcome data, overall performance was not influenced by treatment or gender. The absence of statistical difference can be related to the period under trial (127 d), that is, there was enough time for pigs to overcome a temporary setback before reaching market weight. A reduction in weight gain among regrouped pigs was either not detected [22] or only evident when regrouping was coupled with another stressor such as limited feeding, reduced space, or shipping [23]. Another possible explanation for the absence of statistical difference could be related to familiar environment. Prunier et al. [24] reported that the familiarity of a pen has a greater effect on growth than the identity of pen mates in newly weaned pigs. In this study, weaned pigs were more stressed by shifting to new locations than by regrouping. In the present study, the pigs shifted to different pens were located within the same shed and very close to each other (equal temperature conditions, light and dark cycle, noise, smell, etc.). Moreover, the pens did not have concrete walls, allowing visual and physical contact (nosing, play, non-agonistic contact) with the pen next to it. These, in addition to partial mix of animals, may have mitigated the impact of regrouping on the overall performance parameters.

In contrast, analysis based on period showed that “mixing” influenced feeding behavior. During the mixing of females, barrows spend more time at the electronic feed station (ATES = 1.30 h *vs*. 1.12 h). However, during the mixing of barrows, the females presented a higher feed intake than the barrows (ADFI = 3.27 *vs*. 3.07 kg). Considering that ATES was measured by the amount of time the head was in the feeder hole and that the ADFI was not affected during period–II, it is possible that the pigs were not essentially eating. Based on the findings by Montoro et al. [3], wherein the effect of mixing on productive performance of grower-finisher pigs was investigated, it is possible that mixed pigs could have used the feeder as a “hide” to protect themselves from an attack (submissive behavior) or control the resources (dominant behavior) before consuming the feed. This possibility can be considered based on the fact that the areas with the highest occupancy and incidence of agonistic interactions during the experimental period were QO—2 and QO—1, which are the areas of the electronic feeder and entrance to the pen, respectively.

Furthermore, previous research reported that fighting *per se* may not be the cause of reduction in performance following mixing; however, the stress of threats and submission, which alters the group performance after fighting is over may be [5, 7]. In the present study, the reduction in ADFI and ATES suggests a behavioral change during mixing periods, such as failure to eat. However, as the impact on performance parameters was not observed, with partial regrouping and within a considerable range of days, it can be considered that such strategies could mitigated the stress effects of mixing.

### Sampling methods after mixing

In the present study, three sampling methods were used to collect information during the experimental period to map the frequency and intensity of agnostic interactions during mixing and the differences between gender and areas of greater conflict within the pen. Treatment effect during period–I was not considered because pigs in both groups were subjected to identical conditions (exit from weaning facilities and regrouping to growth–finish period), that is, all the groups presented equal values of negative interactions (Table 3).

Post-mixing aggression occurs as a means of establishing a social hierarchy amongst unfamiliar conspecifics [4]. Time spent fighting was greater among the mixed pigs than the control pigs. According to Desire et al. [25], the ability of a group to form a lasting dominance hierarchy is necessary for long term group stability, and there is evidence that aggressive engagement soon after mixing can improve productivity and reduce chronic aggression over the growing-finishing period. However, avoiding aggression during acute post-mixing phase tends to only delay aggression [25]. According to observations performed during 72-hours after mixing (period II and III), “agonistic interactions”, “lying laterally and sternly”, and “exploring enrichment material” were observed more frequently in the SS group, whereas the highest frequency of “other”, “exploring the pen” and “eating” behaviors were observed in the CT group. Camerlink et al. [26] reported that a good strategy for a pig to avoid fighting is to remain in a lying position, and this may explain why the SS group had a higher frequency for lateral and sternal recumbency than the CT group. An alternative explanation for this is that mixed groups expended more energy in agonistic behavior than that by the CT groups; therefore, they required more rest [26].

According to the results of the present study, a reduction in the number of agonistic interactions was observed in the first 24 hours. Moreover, as the animals reached their slaughter weight, the number of conflicts (agonistic interactions and number of lesions) when the stress factor was applied also reduced considerably, suggesting that hierarchical restructuring occurred very quickly. Furthermore, during period–II, barrow pigs in the SS group initiated most of the agonistic interactions and the females were mostly receivers. For period—III, the highest number of agonistic interactions was observed in the SS group, but no differences were observed between the genders, that is, barrows and females had the same number of author and receiver positions. Notably, in the first case (period II–females’ mix), the new animals were females, whereas in the second case, the new animals were barrows (period III–barrows’ mix); this is something to discuss. Scheffler et al. [17] reported similar results and suggested that a stable dominance hierarchy is established within 48 h after regrouping, suggesting that activities within a pen return to normal after the development of a stable order. Furthermore, males of many species typically show higher levels of overt territorial aggression (attacks, bites, fights) towards an unfamiliar intruder than females [27]. This finding does not suggest that females are not aggressive; in contrast, it suggests that even though females are aggressive, they typically show a predominance of non-overt dominance-related aggression and few or no direct attacks. Consequently, females are underrepresented in laboratory investigations of aggressive behavior that typically focus on overt aggression [27].

Contrary to the initial hypothesis of the study, wherein a greater number of “positive interactions” (PI) within the control group (sniffing, nosing, and licking) was expected, no statistical difference was found between treatments during the three periods. The absence of a statistical difference can be related to the level of excitement and fighting that took place in neighboring pens, and this could have affected the CT group. In the current study, the CT group presented a high level of pen exploration and other behaviors, demonstrating a constant level of activity during the mixing periods in the adjacent pens. Düpjan et al., [28] reported that the general excitement during moving and mixing contributed to overall level of aggression. Control group may have reduced level of positive interactions because they were stimulated by excitement and fighting at nearby pens. These findings could reinforce the hypothesis that the general level of excitement and stress within the shed can affect even pens that have not been mixed. Furthermore, even with the high frequency of the behavior observed in the CT group (period–III), no statistical differences were found in the performance parameters between treatments (performance and feeding behavior results), suggesting that the animals were just handling the feed and not essentially consuming it.

### Injury score

Aggression between unfamiliar pigs at mixing is a serious animal welfare problem in commercial farming [6]. According to previous studies, lesion score (LS) methodology provides a rapid means of estimating aggressive behavioral phenotypes when reference is made to both the number and location of lesions [4]. In the present study, LS methodology was applied after three consecutive regroupings (period I, II and III), to evaluate the differences in the number of lesions between treatment and gender.

As expected, compared with CT group, the SS group presented the highest number of lesions, located primarily in the frontal region (head, ear, and middle) and also in the posterior region. Fighting, pigs attempt to target the head, neck, and ears of their opponent using bites and slashes from the canine teeth [29], resulting in the accumulation of superficial skin lesions predominantly in the front third of the body and in the flanks, when delivered in a reverse parallel posture [4]. Furthermore, lesions in the anterior portion of the body are predominantly associated with reciprocal fighting, and lesions in the posterior portion of the body are predominantly associated with receipt of non-reciprocal aggression and receipt of bullying behavior [4, 25].

According to our results, most of the statistical difference in the lesion score was observed during period—II, when the SS group was undergoing the mixing of females and the hierarchy was already well established in the CT group. During period III, even with the mixing of barrows, there was a reduction in the number of lesions observed, and large part of the observed regions did not indicate difference between treatments. Some studies address the possibility of animals developing strategies to avoid conflict and even the possibility of dominance maintenance by individuals at the top of the hierarchical chain when they changed pens [4, 30]. According to Desire et al. [25], aggressive animals fight amongst themselves in certain pens, whereas less aggressive animals are able to avoid fighting entirely, indicating the progressive reduction in the number of injuries.

For analysis based on gender, the only difference observed were during period—III in which barrows had a higher lesion score for regions LE and LH compared to that of females. Furthermore, gender differences in aggression were based on the type of aggression, with the males showing more overt aggression and the females showing more non-overt aggression to an unfamiliar same gender intruder [1]. Overt aggression can result in injury and often causes the loser to leave the territory of the winner, whereas non-overt aggression typically leads to the formation of dominance hierarchies that regulate the social dynamics of a group, for example, the controlling of access to critical resources, while enabling cohabitation among group members [27]. However, according to Tong et al. [12], the time required to achieve social stability in pigs after mixing was lower for females than for males, leading to less agonistic interactions. For other periods, no statistical differences were observed and other studies were unable to detect a behavioral difference between gender in the aggression of growing pigs [11]. Therefore, future studies on the possible differences in the aggression patterns of males, barrows, and females that have or have not undergone previous stress (intact litters or mixed) is needed for possible applications as farm management strategies.

### Physiological measures

According to neuroendocrinology, stress is any stimulus that provokes the release of adrenocorticotropic hormone (ACTH) and adrenal glucocorticoids, and can be indicated by an increase in peripheral cortisol concentration [31], an increase in the circulatory segmented: lymphocyte ratio (Seg:Lynf) [32], impaired immune response [33], and elevated body temperature [34]. According to our results, all pigs started the experimental period under the same physiological conditions. For the final samples from the CT group presented higher values of Seg:Lynf compared to those from the SS group (1.24 *vs*. 1.04). Both treatments had values above the reference intervals (Seg:Lynf; 0.38–0.85) [35].

Previous studies that showed differences in hematological parameters and cortisol collected samples hours after the stress factor was applied. Montoro et al. [3] and Foister at al. [4] collected sample within a short period after mixing, and regrouping increased plasma cortisol concentration at 24 and 8 h, respectively. Furthermore, according to [31], the acute response to mixing that occur within hours decreases over time and repeated regrouping, suggesting a habituation to the stressful situation. Therefore, the influence of a chronic intermittent stressor on hematological parameters and cortisol may have increased influence when the stressor is likely to be more aversive. However, despite its wide use, cortisol concentration is influenced by many factors and could limit its use as a stress biomarker [36]. The average concentration of cortisol in pigs decrease with age, reaching a stable profile around 20 weeks of age, when the levels were approximately 37% lower compared to that at 12 weeks of age [37]. In addition, gender is another source of variation, with concentration in males being approximately 15% higher than that in gilts [38]. All these factors linked to cortisol as biomarker, which are associated with the stress factor’ intensity, may have contributed to equalize the samples between treatments, thereby leading to the absence of statistical difference.

For gender, basal samples of females presented higher values of mean corpuscular volume (MCV) (60.62 *vs*. 58.71 fL) than those of barrows, and the final samples of barrows presented the highest hemoglobin (Hb) count (13.28 *vs*. 12.70 g/dL) compared to that of females. Hematologic and biochemical reference intervals depend on many factors, including environment and age, and the results can significantly change management and breeding in pig farms [39]. Although the samples differed from each other statistically, the values found for MCV and Hb were within the reference range for hematological parameters of pigs, which are MCV (47.7–63.0 Fl) and Hb (11.1–14.4 g/dL) [35].

One of the objectives of the present study was to assess the accumulated impact of successive regrouping of pigs during growing and finishing period; hence, analysis of cortisol in the hair and blood are shown to be efficient. Thus, according to the Seg:Lynf results, the animals went through a period of stress, even the blood cortisol values remained unchanged. In the future, it would be better to collect samples hours after mixing to analyze different biomarkers, such as chromogranin A (CgA), acute phase proteins (APP), and immunoglobulin A (IgA).

## Conclusion

The results of the present study showed that repeated regrouping significantly affected social and feeding behavior without severely altering performance and physiological parameters. Furthermore, based on the gender that was mixed, different patterns of social and feeding behavior, agonistic interactions, and injury score between barrows and females were observed. This study provides an understanding on the impact of hierarchical structure and gender differences in pigs, and this knowledge can be used to improve swine productivity and welfare.
