# Correction: Mixed management in growing and finishing pigs: Differences between gender and their impacts on behavior, growth performance, and physiological parameters

**DOI:** 10.1371/journal.pone.0294103

**Published:** 2023-11-02

**Authors:** Angela Cristina da Fonseca de Oliveira, Leandro Batista Costa, Saulo Henrique Weber, Yuliaxis Ramayo-Caldas, Antoni Dalmau

There are errors in the Funding section. The correct Funding statement is: This study was funded by the Spanish Program of Research across the Ministry of Science and Innovation of the Spanish Government (MICYN) with the code PDC2021-121269-100. The funders had no role in study design, data collection and analysis, decision to publish, or preparation of the manuscript.
